# Blueprint for nanoscale NMR

**DOI:** 10.1038/s41598-019-43404-2

**Published:** 2019-05-06

**Authors:** Ilai Schwartz, Joachim Rosskopf, Simon Schmitt, Benedikt Tratzmiller, Qiong Chen, Liam P. McGuinness, Fedor Jelezko, Martin B. Plenio

**Affiliations:** 1 Institute of Theoretical Physics and IQST, Universität Ulm, Albert-Einstein-Allee 11, 89081 Ulm, Germany; 2 NVision Imaging Technologies GmbH, Universität Ulm, Albert-Einstein-Allee 11, 89081 Ulm, Germany; 3 Institute of Quantum Optics and IQST, Universität Ulm, Albert-Einstein-Allee 11, 89081 Ulm, Germany

**Keywords:** Quantum mechanics, Quantum metrology

## Abstract

Nitrogen vacancy (NV) centers in diamond have been used as ultrasensitive magnetometers to perform nuclear magnetic resonance (NMR) spectroscopy of statistically polarized samples at 1–100 nm length scales. However, the spectral linewidth is typically limited to the kHz level, both by the NV sensor coherence time and by rapid molecular diffusion of the nuclei through the detection volume which in turn is critical for achieving long nuclear coherence times. Here we provide a blueprint supported by detailed theoretical analysis for a set-up that combines a sensitivity sufficient for detecting NMR signals from nano- to micron-scale samples with a spectral resolution that is limited only by the nuclear spin coherence, i.e. comparable to conventional NMR. Our protocol detects the nuclear polarization induced along the direction of an external magnetic field with near surface NV centers using lock-in detection techniques to enable phase coherent signal averaging. Using the NV centers in a dual role of NMR detector and optical hyperpolarization source to increase signal to noise, and in combination with Bayesian inference models for signal processing, nano/microscale NMR spectroscopy can be performed on sample concentrations in the micromolar range, several orders of magnitude better than the current state of the art.

## Introduction

Nuclear magnetic resonance (NMR) and magnetic resonance imaging (MRI) are technologies whose applications in organic chemistry, biology, medicine and material science have enabled fundamental scientific breakthroughs and continue to be drivers of scientific and technological progress^1^. Despite these successes, it is recognized that nuclear magnetic resonance applications have limitations due to the minute nuclear magnetization of analytes which leads to limited sensitivity in comparison to other analytic techniques such as mass spectrometry.

Strategies that are being pursued to overcome this challenge include an evolution towards larger applied magnetic fields which improves sensitivity due to the resulting increase of thermal equilibrium polarization and signal frequency^2^ but comes at the cost of growing size, purchase and operating costs of these devices, which in turn limits portability and challenge their integration with desired applications. More compact magnets lead to smaller usable detection volumes and thus limit sensitivity. A promising alternative strategy is the reduction in size of the radio frequency coils used to excite and detect the NMR signals^3^ as this results in a sensitivity enhancement with decreasing coil-diameter and promises the development of portable on-chip NMR spectrometers^4^. Limitations and challenges in this approach include the homogeneity of the system which limit resolution and the thermal noise in the readout coil, i.e. thermal Johnson noise, which, together with the low sample volume, limits sensitivity. A further avenue towards improved NMR sensitivity is to increase the nuclear spin polarization beyond its thermal equilibrium value by means of techniques such as dynamical nuclear polarization^5^. Despite promising results, the integration of these approaches with NMR involve significant challenges, as they typically require low temperatures and dissolution of the sample - significantly reducing its concentration.

Addressing these challenges in a single device to achieve simultaneously improved sensitivity, ideally in the micromolar regime, portability and the ability to vary sample volumes from the nano- to the millimeter scale would decisively enhance a broad range of applications thus offering the potential for new ground breaking insights. These include NMR studies of single cells and neurons^6^, surfaces, surface chemistry and catalysis at smallest volumes^7^, and on-chip NMR based metabolic fingerprinting with applications in personalized medicine^8,9^. Moreover, if such NMR detection can be performed in ultra-low magnetic fields, information-rich spectra can be obtained due to the J coupling in this regime, as shown in the field of zero and ultra-low field (ZULF) NMR^10–13^.

Here we present a novel physical platform for NMR detection that we show is capable of overcoming these challenges for samples ranging from the nano- to the millimeter-scale using nitrogen vacancy (NV) centers in diamond as a well-established quantum sensor^14–20^. We introduce an NMR protocol that permits spectroscopy of such volumes with chemical resolution and micromolar range sensitivity and demonstrate signal processing algorithms that allow for a significant reduction in signal acquisition time, thereby yielding sample analysis with dramatic speed-up. The feasibility of this approach is experimentally demonstrated by detecting magnetic signals applied to a single NV center in diamond. The signal dynamics are obtained from atomistic simulations of a diffusive nanoscale nuclear sample, with a signal intensity scaled to correspond to an NV depth of 6.2 nm.

## Results

### Background and key design elements

High-resolution NMR spectroscopy makes use of several properties of bulk matter in resolving chemical shifts and J-couplings for molecular structure determination. The rapid molecular diffusion and rotation leads to the suppression of internuclear interaction down to the Hz-level while not limiting the signal coherence due to the large volume from which the signal is collected. Furthermore, for bulk samples, the thermal polarization (scaling with the sample volume *V*) greatly exceeds the statistical polarization fluctuations (scaling with *V*^1/2^). This allows on the one hand for the controlled initialization of the signal and therefore phase coherent signal accumulation resulting in a rapid growth of the signal to noise ratio (SNR) and on the other hand for long signal coherence times and therefore high spectral resolution.

However, for (1 *μm*)^3^ of water in a 1 Tesla field, the statistical polarization of the hydrogen nuclei is comparable to the thermal polarization and becomes dominant at the nanoscale. This observation has motivated successful experimental efforts towards NMR detection of statistical polarization of nanoscale samples^21^. On the other hand, the stochastic nature and random phase of the observed statistical polarization prevent phase coherent signal averaging and the impact of diffusion limits signal coherence time and thus spectral resolution^22^. Overcoming these limitations calls for new modes of observation. Here we will consider, perhaps counter-intuitively, the detection of the signal originating from the thermal polarization, even for nanoscale samples for which the statistical component is expected to dominate. We use three key features to compensate for this apparent shortcoming, namely, (i) the signal phase can be controlled by an initializing *π*/2-pulse to allow for phase coherent accumulation across subsequent measurements, (ii) because the thermal polarization component is uniform across the entire sample beyond the immediate detection region, the signal coherence time becomes essentially independent of diffusion allowing for high spectral resolution, (iii) and for the same reasons the signal is uniform across the sample which allows for the use of multiple NV-centers for simultaneous signal acquisition thus further improving SNR.

The platform and protocols described in the following leverage the unique characteristics of color centers in diamond^23^ to make use of these three key features. First, we use optically detected magnetic resonance in either individual or ensembles of color centers^15,24^ to detect small magnetic fields emanating from the sample which, by making use of a recently developed lock-in technique, Qdyne, allows for spectral resolution in the *μ*Hz range^25–27^. This substitutes the electrical detection via rf-microcoils which is accompanied by thermal Johnson noise by optical detection which is only limited by the non-thermal photon shot noise. Secondly, the ability to bring color centers to within nanoscale distance of the sample allows for their use as a source of nuclear hyerpolarization even under ambient conditions using laser induced polarization of the electron spin native to the color center and the subsequent microwave assisted transfer to the sample nuclei^28–30^. This obviates the need for a strong magnetic field and holds the potential for an orders of magnitude increase in signal strength, thus bringing sub-millimolar sensitivities into reach while at the same time reducing nuclear spin polarization fluctuations induced by diffusion. Thirdly, in order to reduce averaging times required for achieving sub- millimolar sensitivities, we employ signal processing methods based on Bayesian inference algorithms that allow for orders of magnitude reduction of measurements required for the identification of signal components due to chemical shifts. The remainder of this work will describe these key elements and present theoretical and experimental results that demonstrate the feasibility of the approach.

### Qdyne for nuclear magnetic resonance

The recently developed Qdyne method introduces a quantum lock-in spectroscopy technique^25–27^ whose spectral resolution is independent of the sensor coherence time. Using Qdyne, a coherent external oscillating radiofrequency (RF) field, could be measured with a spectral linewidth of 607 *μ*Hz^25^, thereby making the technique promising for realizing true nanoscale NMR via shallow NV centers.

In Qdyne, the sensor qubit is tailored to collect a signal that depends not only on the amplitude and frequency of the detected field, but also on the phase with respect to the start of each measurement. Performing *N* measurements each of length *T*_*L*_, a different phase is accumulated in each measurement due to the difference between *T*_*L*_ and period of the oscillatory field. As shown in ref.^25^, for an XY8 measurement sequence when the excitation and detection *π*/2 pulses are perpendicular to each other, the detected signal is given by

1 $$P=\frac{1}{2}\,\sin \,(\frac{4k{\tau }_{m}}{\pi }\,\cos \,(\delta t+\varphi ))+\frac{1}{2},$$

where *k* is the total interaction strength, ${\tau }_{m}$ the interaction time, $\varphi$ is an arbitrary initial phase of the RF field, and *δ* denotes the frequency of the accumulated phase.

As with most NV sensing schemes, when using a shallow NV for detection, due to the small number *N*_*I*_ of spins in the vicinity of the NV, the signal detected by Qdyne is dominated by the statistical polarization of the nuclear spins in the sensing volume - ${B}_{rms}(t)={\sum }_{i}\,{A}_{x}^{i}(t){I}_{x}$, with ${A}_{x}^{i}(t)$ denoting the coupling of the NV center to the *i*-th nuclear spin.

Diffusion of molecules into and out of this volume leads to random fluctuations of the detected signal, which is governed by a correlation function $\langle {B}_{rms}(t){B}_{rms}(t+\tau )\rangle ={B}_{rms}^{2}\,\exp \,(\,-\,\tau /{\tau }_{c})$, where ${\tau }_{c}$ is the correlation time. Thus, the phase $\varphi$ in Eq. (1) becomes a stochastic variable, $\hat{\varphi }(t)$, which denotes the instantaneous phase of the statistical nuclear spin polarization within the NV detection region. Some fluctuations in $\varphi$ are still expected due to spatial deviations of the molecule locations, however, this is expected to be negligible even for shallow NVs. *k* in equation (1) would also fluctuate due to the density and spin fluctuations, but to a much lesser extent than $\hat{\varphi }(t)$.

**Figure 1:**
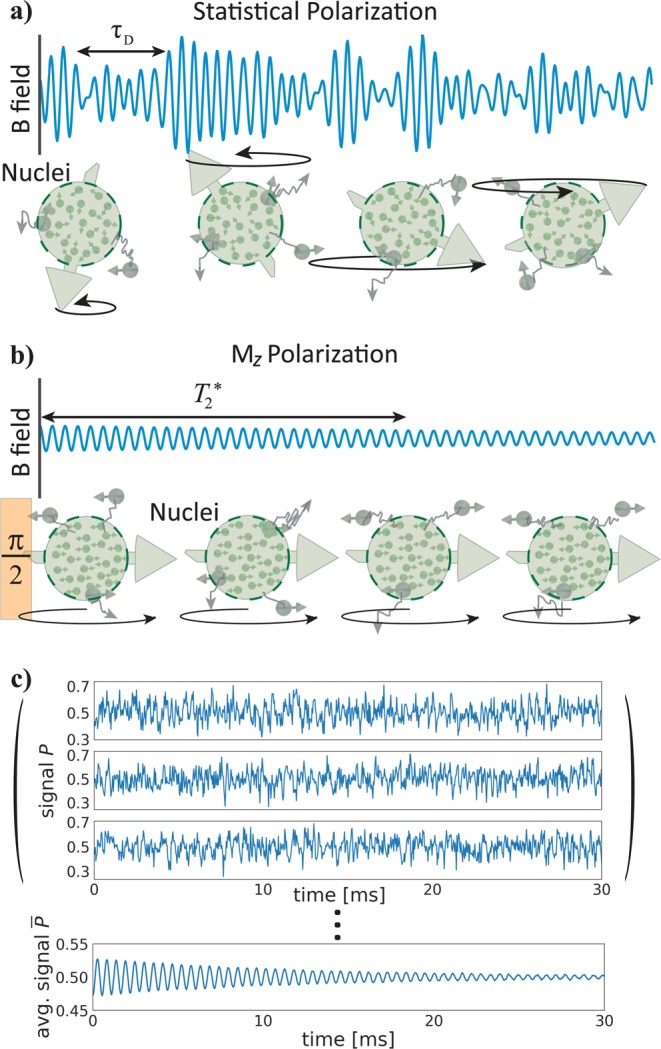
(**a**) Illustration of the Qdyne signal and the detected statistical magnetization by the NV center. The diffusion of nuclear spins in or out of the NV detection range leads to rapid fluctuations of the detected phase, introducing a short signal coherence time-scale, ${\tau }_{D}$, on the order of the interaction correlation time. (**b**) The adoption of *M*_*z*_ Qdyne enables the detection of the FID signal from the nuclear z magnetization (thermal or hyperpolarized), regardless of the molecular diffusion. (**c**) An atomistic simulation of the time-dependent magnetic field induced by the diffusing nuclear spins on a 6.2 nm deep NV, where the statistical polarization is still clearly stronger than the hyperpolarized ${M}_{z}=0.1 \%$ polarization. However, *M*_*z*_ Qdyne can be averaged over many runs *N*_*m*_, significantly reducing the statistical polarization signal by 1/$\sqrt{{N}_{m}}$ while the *M*_*z*_ signal remains unchanged (due to the same initial phase at every run). The bottom figure shows the averaged signal for nuclear ${T}_{2}=5\,ms$ with ${N}_{m}=1500$, where only the smaller *M*_*z*_ polarization (oscillates between ±0.05) remains visible.

This stochastic variable ties the observed Qdyne signal to the molecular diffusion of the moving molecules, leading to a stringent limitation on the minimal observable line-width, thereby obscuring small but important details such as chemical shift and quadrupole information. See Fig. 1(a) for an illustration of a statistical magnetization detected by a Qdyne measurement, and the diffusion effect on the phase of the detected signal.

To solve this issue, and decouple the Qdyne signal from molecular diffusion, we modify the sequence to detect thermal nuclear magnetization along the z-axis of the applied magnetic field. This modified *M*_*z*_ Qdyne sequence consists of *N*_*m*_ measurements, and, at the beginning of the sequence, a *π*/2 pulse which rotates the nuclear z magnetization to the x-y plane, where it can be detected by the XY dynamical decoupling measurement with the correct filter function, similar to a free induction decay (FID) in traditional NMR. Importantly, at the beginning of each of the *N*_*m*_ sequences, the initial phase of the z magnetization on the x-y plane is known and *identical*. Moreover, as the phase is identical for all nuclear spins across the sample, the diffusion of molecules in or out of the NV center detection region has no effect on the signal phase, and the detected linewidth becomes limited only by the nuclear coherence times, see Fig. 1(b).

In a realistic NMR scenario using a 5–100 nm deep NV center, both the statistical and *M*_*z*_ sample magnetizations contribute to the detected signal, with the statistical polarization generally dominating the z-magnetization. However, the summation of *N*_*m*_ repeated measurements can be used to reduce the statistical signal by a factor of 1/$\sqrt{{N}_{m}}$ due to its random phase and magnitude, while locking-in to the phase of *M*_*z*_-magnetization so as to prevent its cancellation. Thus, especially when combined with hyperpolarization, the detection of *M*_*z*_ magnetization is feasible even with relatively shallow NV centers, and therefore even sub-micron detection volumes. Figure 1(c) shows the Qdyne phase accumulated by the NV in an atomistic simulation of diffusing hyperpolarized ($\langle {M}_{z}\rangle =0.1 \%$) nuclear spins near a 6.2 nm deep NV center. The diffusion coefficient was chosen to be $D={10}^{-12}\,{m}^{2}$/*s*, similar to oil molecules. Clearly the statistical polarization is larger than the hyperpolarized signal in this regime as the FID is not visible. However, when averaging the signal over 300 runs, the smaller *M*_*z*_ polarization can be clearly seen due to the reduction of the contribution from the statistical polarization.

An additional advantage of *M*_*z*_ Qdyne due to the averaging of *N*_*m*_ measurements is better statistical information on each measured point. This improves the low photon collection efficiency inherent in NV-based detection, with the statistical detection process becoming a Poissonian distribution rather than a Bernoulli process. Moreover, as the *M*_*z*_-polarization is uniform across the entire sample, different NV centers now detect the same phase $\varphi$, which allows the measurement to be performed with ensembles of NV centers. The accumulated fluorescence from the different NV centers acts identically to repeated measurements *N*_*m*_ of the single NV center, i.e. improving statistical information and averaging out the statistical polarization. Thus, the number of statistical averages is given by $N={N}_{NV}\times {N}_{m}$, where *N*_*NV*_ is the number of NV centers used as sensors.

**Figure 2:**
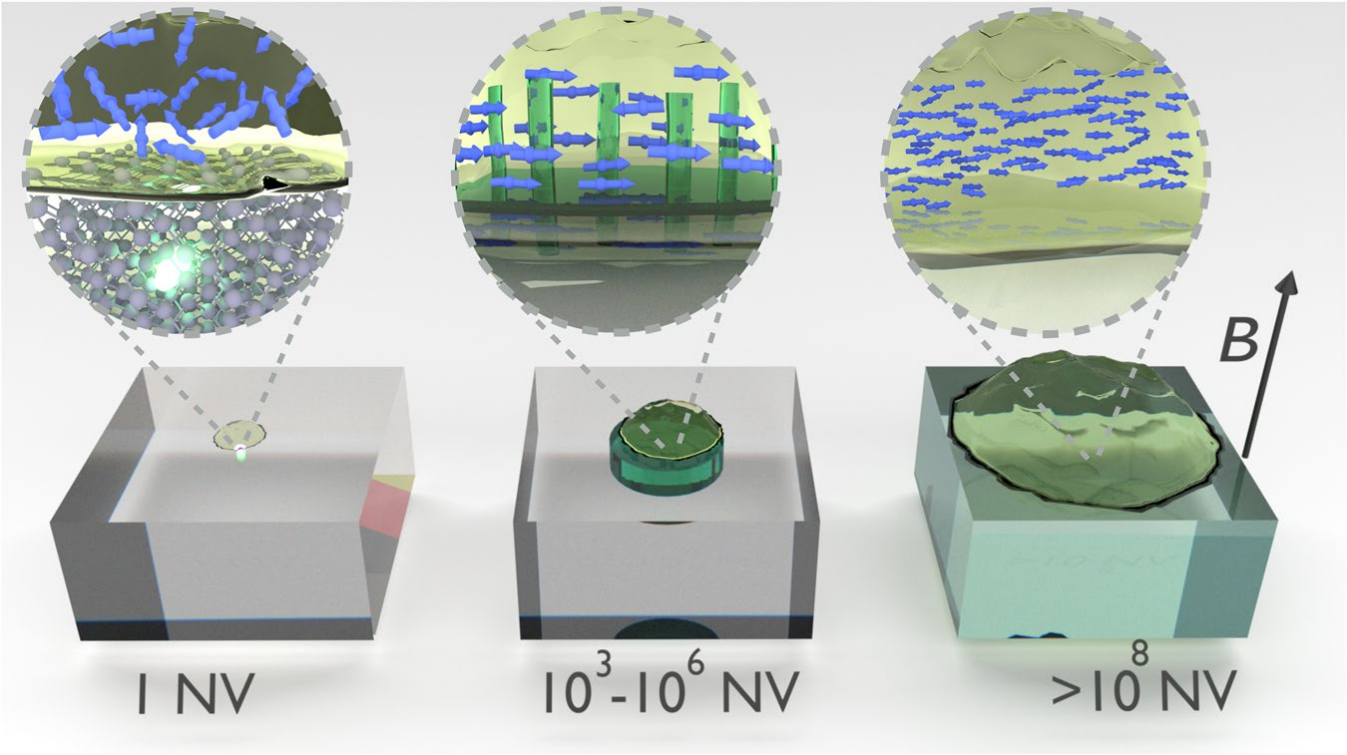
Illustration of the three different regimes made possible by *M*_*z*_ Qdyne. On the r.h.s. is the “classical” regime, where a macroscopic diamond with densely packed NV centers senses the thermalpolarization, providing a substitute to traditional NMR micro-coils. In the middle figure, using the NV center ensemble in a dual role- polarizing the nuclear spin bath to increase the nuclear signal and detection of the NMR signal, thus termed Hyperdyne, enables superb sensitivities in the nano- and micro-scale regimes (the illustration includes a nanostructured diamond for enhanced polarization efficiency). Hyperdyne can be pushed to the extreme nano-scale limit, using a single NV center (l.h.s.) albeit at a significantly reduced polarization/sensitivity efficiency.

*M*_*z*_ Qdyne unlocks the potential of NV-based NMR, allowing for volumes ranging from nanometric to macroscopic scales. However, as the nuclear thermal polarization is very weak, especially at lower magnetic fields, achieving a good SNR in this regime requires macroscopic diamonds with densely packed NV centers and roughly 1 *μl* samples (1 mm^3^) for practical NMR applications (r.h.s. of Fig. 2). Pushing the limits of this regime to even large micro-scale would require averaging of several hours of measurement time for acquiring a sufficient SNR, due to the small signal produced by thermal polarization. Thus, in this regime the NV ensemble in the diamond serves as a “classical” macroscopic NMR sensor, similar to currently used micro-coils, albeit with the advantage of different noise processes which could lead to improved sensitivity. To push the application into the micro- and nano-scale regimes, the *M*_*z*_ polarization needs to be enhanced, in a manner which still allows fast repetitive measurements. Fortuitously, optically polarized NV centers have been demonstrated to be superb polarization sources for nuclear spin hyperpolarization either inside the diamond^31–35^ or in external molecules^28–30^. Thus by using the NV centers in a dual role of hyperpolarization sources and NMR detectors, with shallow NVs used for polarization and deeper ones for detection, the NMR SNR of each measurement can be increased over 10,000-fold, without the need for shuttling between polarization and detection zones and without suffering other detrimental side-effects of dissolution DNP (e.g. cooling the sample to $T=1\,K$, reduction of analyte concentration upon dissolution). Thus, using interleaved hyperpolarization/detection sequences on the NV centers (the Hyperdyne protocol) one can achieve true NMR applicability on the (sub)micronscale, see middle of Fig. 2. Note that the combination of hyperpolarization and spin-based readout has been shown to be instrumental in ZULF NMR applications^10–13^. The Hyperdyne protocol builds on these developments and enables performing for the first time both the hyperpolarization and mesoscopic NMR detection using the same electron spins. This is in contrast to the recent use of traditional DNP techniques based on admixed electron spin radicals in combination with NV center based detection^36^ which is limited in achievable degree of nuclear polarisation by the electron radical thermal polarization.

On the extreme nanometric scale (l.h.s. of Fig. 2), one may implement Hyperdyne NMR with a single NV center. However, the single NV center needs to be close to the surface for sufficient efficiency in the hyperpolarization cycle, which leads to the disadvantage that many of the polarized nuclei will diffuse outside the small NV detection region, and thus produce a much smaller net gain in the NMR sensitivity.

In the next two sections we first provide expressions for the signal to noise ratio for large numbers of detection events, either due to high detection efficiency or large number of phase coherent averages that apply to a standard Fast Fourier Transform (FFT) analysis and present the results of the experimental detection of simulated signals by means of an NV center. In the following section we will then describe Bayesian inference methods to improve signal detection from noisy measured data for situations in which signals are so noisy that standard FFT analysis is not able to extract frequency information from data. We will demonstrate that Bayesian inference methods are capable of extracting information from significantly more noisy signals and thus allow for orders of magnitude reduction in measurement time.

### Verification and FFT signal analysis of hyperdyne

**Figure 3:**
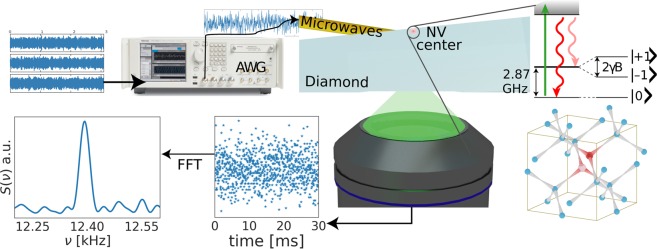
Experimental verification of the Hyperdyne method. Using an arbitrary waveform generator (AWG), the simulated magnetic field (*N*_*m*_ = 1000) was applied on a single NV center by a current carrying wire, with the setup of ref.^25^. Taking a Fourier transform of the summed photon counts, the *M*_*z*_, diffusion independent signal can be clearly seen, producing a peak with 170 Hz linewidth (limited only by the 5.6 ms length of the simulated signal). A simulated signal with only statistical polarization produces no peak, and the diffusion limited signal would be over 10 kHz.

To experimentally verify the Hyperdyne protocol, the magnetic field produced by an ensemble of nuclear spins as calculated by atomistic simulations was inputed into an arbitrary waveform generator and applied to a single NV center by a current carrying wire (see ref.^25^ and the methods section for experimental details) for ${N}_{m}=1000\,{\rm{runs}}$ as shown in Fig. 3. While in each individual run the number of photons detected in each measurement was typically 0 or 1, due to the summation of the *N*_*m*_ runs, a much better statistics of the photon count was achieved, even given the low detection probability. A measured signal due to the *M*_*z*_ polarization is clearly visible with 170 Hz linewidth, limited only by the 5.6 ms length of the detected signal. For comparison, with the chosen diffusion parameter the linewidth due to the statistical polarization would exceed 10 kHz.

We proceed with theoretically analysing the behaviour of the Hyperdyne signal. For shot noise limited detection,

2 $$SNR\propto \sqrt{{N}_{{\rm{Phot}}}}k{\tau }_{m}\propto \sqrt{N}\rho {P}_{n}{\tau }_{m},$$

with ${N}_{{\rm{Phot}}}\propto {V}_{s}$ being the number of detected photons, ${\tau }_{m}$ the length of a single XY measurement, *V*_*s*_ the total detection volume, *k*(${\tau }_{m}$) the interaction strength (time) for an individual NV center, $N={N}_{m}{N}_{NV}$ the number of independent measurements (which is the product of *N*_*m*_ runs with *N*_*NV*_ NV centers), $\rho$ the nuclear spin concentration and *P*_*n*_ the average polarization. There are two regimes to be considered when aiming to maximize SNR in a given total experiment time here. Firstly, when ${\tau }_{m} < \,{\rm{\min }}\,({T}_{2}^{NV},\pi /4k)$ it is most advantageous to increase ${\tau }_{m}$ while keeping *N*_*m*_ constant, i.e. increase the time over which the signal is accumulated coherently. In this case the total measurement time $T={N}_{m}{\tau }_{m}$ scales as the first case of eq. (3). If however, ${\tau }_{m}={T}_{2}^{NV}$ we cannot increase ${\tau }_{m}$ any further without suffering an exponential in ${\tau }_{m}$ loss in signal (for ${\tau }_{m} > \pi$/4*k* we lose the ability to identify the phase). Hence we are reduced to increase *N*_*m*_, that is averaging over independent runs. In this case the total measurement time $T={N}_{m}{\tau }_{m}$ scales as the second case of eq. (3) benefiting more from an increase in polarization of the sample.

3 $$\frac{1}{T}\propto \{\begin{array}{ll}\sqrt{{V}_{s}{\rho }_{NV}}\rho {P}_{n} & {\rm{if}}\,{\rm{increasing}}\,\,{\tau }_{m} < \,{\rm{\min }}\,({T}_{2}^{NV},\frac{\pi }{4k})\\ {V}_{s}{\rho }_{NV}{\rho }^{2}{P}_{n}^{2} & {\rm{else}}.\end{array}$$

where *T* is the total measurement time for achieving a fixed SNR value, ${\rho }_{NV}$ is the NV concentration, and enlarging *V*_*s*_ is assumed to be achieved by increasing the surface cross section, thereby scaling linearly with the number of NV centers.

**Figure 4:**
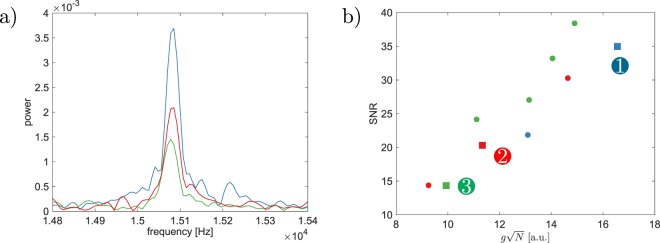
(**a**) The Fourier transform of the NV Hyperdyne signal with three different parameters for the diffusing nuclear spins. The SNR mainly depends on the signal amplitude within each XY measurement $g=k{\tau }_{m}$ and the number of measurements *N*. (**b**) Scaling of the SNR with *g*$\sqrt{N}$ for numerous parameter configurations, noting specifically the three parameters from (**a**), curve expected to be linear by Eq. 2. The parameters are ${P}_{n}=0.1 \%$, $\rho =5\,M$, $N=1600$, ${\tau }_{m}=4.1\,\mu s$ (data set 1), ${P}_{n}=0.5 \%$, $\rho =5\,M$, $N=30$, ${\tau }_{m}=4.1\,\mu s$, (data set 2) and ${P}_{n}=0.05 \%$, $\rho =150\,mM$, $N=400$, ${\tau }_{m}=32.8\,\mu s$ (data set 3).

Figure 4(a) shows the Fourier transform of the acquired *M*_*z*_ Qdyne signal by a 6.2 nm deep NV center for three scenarios with different polarization, molecular concentration and number of measurements *N*. All scenarios were produced by atomistic simulation of the detection process for diffusing nuclei at the density of water. One can see a difference in the SNR between the three scenarios, due to a difference in $g=k{\tau }_{m}$ and *N*. As shown in Fig. 4(b), the scaling of the SNR is proportional to *g*$\sqrt{N}$, as expected from Eq. 2.

As noted above, the statistical polarization can be allowed to be larger than the *M*_*z*_ polarization as it is reduced by the averaging of the signal over *N*_*m*_ runs. The statistical polarization does limit the accumulation time in the XY sequences for shallow NV centers, as the condition ${\gamma }_{e}{B}_{rms}{\tau }_{m} < \pi$/2 needs to be fulfilled to ensure that the *M*_*z*_-signal is not fully randomized by the statistical polarization. As hyperpolarization enhances the *M*_*z*_ to statistical polarization ratio, a larger *M*_*z*_ signal can be accumulated by shallow NV centers, enabling the sensing of nano-scale volumes. It is important to note that the shot noise in the detection process scales as $\sqrt{N}$, as does the statistical polarization signal, which in turn implies that the fluctuations due to the statistical polarization signal are never larger than the shot noise (including the pre-factor due to ${\gamma }_{e}{B}_{rms}{\tau }_{m} < \pi$/2), and are typically negligible.

It is interesting to compare our scheme to microcoils. When scaling the diamond to the macroscopic regime (e.g. 1 *μ*l), the expected sensitivity will be similar to that achieved with state of the art micro-coils. However, due to the ohmic contribution to the noise becoming dominant at small diameters^37^, microcoil sensitivity per unit volume starts scaling as 1/$\sqrt{d}$ instead of 1/*d* when *d* < 100 *μ*m, where *d* is the coil diameter, equivalent to 1/${V}_{s}^{1/4}$^3^. Therefore, even for large micrometric samples NV detection may become superior to microcoil detection, even without combining with hyperpolarization.

### Advanced signal analysis of hyperdyne: From FFT to hierarchical Bayesian models and Monte Carlo inference

The measured Hyperdyne photon count signal **D** is acquired by photo detectors with a detection scheme similar to that of ^38^ and suffers from several loss sources that make the acquired signal very noisy. On the one hand the detection is extremely lossy, leading to a very sparse time series of photon counts with less than a detection event per signal period. On the other hand each NV emits with a finite probability a photon in the $|\,-\,1\rangle$ state, leading to just a small net difference in detection probability *p* between $|0\rangle$-state (${p}_{|0\rangle }\approx 4.0 \%$) and $|\,-\,1\rangle$-state (${p}_{|-1\rangle }\approx 2.5 \%$). In the framework of Fourier NMR spectroscopy, this setting leads to rapidly decaying SNR. Of note, the detection efficiency can be significantly increased to almost 100% by using single-shot readout.

In this challenging, low SNR settings, approximating the parameters of an underlying, hidden model by Bayesian inference has shown great benefit in other experimental scenarios (e.g. in astro- or particle-physics^39,40^ and recently in NV center measurements^41^). In^42,43^ and more recently^25^ it was mentioned, that Fourier analysis is not necessarily optimal in terms of precision and other estimation methods, such as maximum likelihood, can be used.

Similar to FFT the hierarchical Bayesian method operates on the raw signal vector **D** without any preprocessing or reconstruction, but at the same time reducing the measurement time by at least one order of magnitude in typical settings. It relies on a probabilistic graphical model (PGM) capturing the hierarchical nature of the Rabi oscillation, photon emission and detection. This parametric model allows to incorporate prior knowledge of the problem into the analysis of the sparse signal. By using Bayesian inference NMR spectroscopy can be interpreted as fitting the distribution of parameters of an underlying harmonic model. The fit is guided by measured data **D** and an informed choice of priors of the parameters ${\rm{\Theta }}$. The priors which go into the population probability *P* in eq. (1) are determined by a normally distributed $g\sim {{\mathscr{N}}}_{\mu }(\mu =4k{\tau }_{m}/\pi )$, the uniform oscillation frequency $\delta \sim {{\mathscr{U}}}_{a,b}({\delta }_{0}-a,{\delta }_{0}+b)$ and an uniform free phase parameter $\varphi \sim {{\mathscr{U}}}_{a,b}([0,2\pi ])$. Descending from *P* the measurement is modeled by $M\sim {{\mathscr{P}}}_{\lambda }(\lambda ={p}_{|-1\rangle }+({p}_{|0\rangle }-{p}_{|-1\rangle })P)$, where ${{\mathscr{P}}}_{\lambda }$ is the Poisson distribution for the photon counts. It’s rate parameter *λ* is determined by the parent emission process. The resulting distributions after the fit are called posteriors. An advantage of the hierarchical model is the ability to directly model the photon counts with a discrete probability distribution, instead of approximating the signal by a continuous distribution. The approximation via a continuous distribution is typically justified by the central limit theorem, which is applicable if ${\rm{\dim }}({\bf{D}})\to \infty$. However in our case, where ${\rm{\dim }}({\bf{D}})\approx {\bf{1}}{{\bf{0}}}^{{\bf{5}}}$ and the assumption of a continuous model constitutes an approximation that would lead to loss of precision and model performance.

**M** is a vector of stochastic random variables (RVs) as it depends on parents in the PGM. The parents could either be constants or random according to a specified probability distribution. The value of the vector is determined by the measured photon counts. It’s up to the inference mechanism to estimate the posterior model parameters ${\rm{\Theta }}=g,\delta ,\varphi$ such, that the posterior distribution approximates the measured values best. The adjustment of the posteriors is done by drawing many samples from a proposal distribution using Markov Chain Monte Carlo (MCMC) and either keeping the current ${\rm{\Theta }}$ with a certain probability if the likelihood of the measured data is increased, or rejecting the sample. The MCMC sampling takes the form of a Markov-Chain which means the position of step *n* + 1 is dependent only upon the position of step *n*, and is otherwise independent of all other steps. The walk around the joint proposal distribution happens in a semi-random manner. The step-size and direction are decided according to specific rules of the sampling method, including randomness (the Monte-Carlo aspect) and gradient-seeking and momentum (Hamilton Monte Carlo^44^) for efficiency. Simpler algorithms like maximum likelihood (ML) or expectation maximization (EM) couldn’t be directly applied, due to the complex nature of the likelihood function. If the MCMC algorithm has converged sufficiently well, the samples drawn approximate the respective posterior distributions of the RVs. The whole procedure can be seen as a stochastic simulation of the experiment and adjustment of the parameters until measured and simulated data is statistically equivalent. To implement the inference algorithm we relied on recent software techniques, which provide easy to use and flexible to program inference algorithms to model complex problems. Practically the performance of the sampling highly depends on it’s initial starting value^45^. Therefore an as good as possible a posteriori (MAP) estimate is used as a starting point. Recently tools for probabilistic programming (PP), automatic differentiation frameworks and advances in MCMC methods made automatic Bayesian inference on PGMs easy to formulate and perform. The tool used in this work is called PyMC3^46^. The framework automatically derives a likelihood function for the model and repeats the sampling and evaluation for a a defined upper bound. This reduces implementation effort and make quick model changes possible.

**Figure 5:**
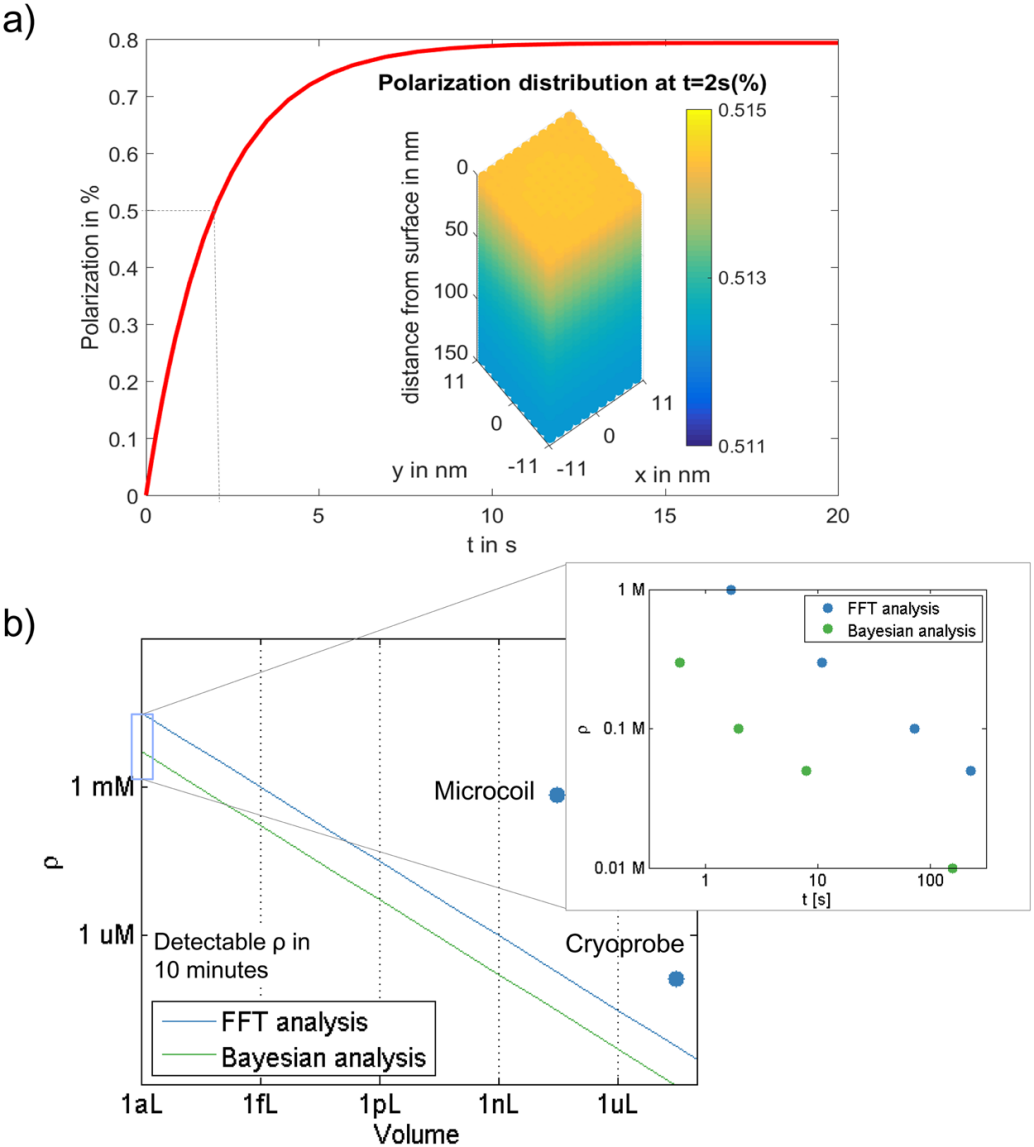
(**a**) Simulation of the polarization buildup for our setup, taking into account the polarization rate, molecular diffusion and nuclear relaxation of ${T}_{1}=2\,{\rm{s}}$. (**b**) The minimal detectable molecular concentration for different volumes using the $\sqrt{N}$ dependence (volume increased by increasing the surface cross-section). A SNR of 10 was chosen as the threshold. Also shown are the best achieved sensitivities for cryoprobes and microcoils^47^. The insert depicts the time required for detection (SNR > 10) of different concentrations for a volume of (100 *nm*)^3^, used for the calculation of the detectable minimal concentration within 10 minute time.

The benefit of a Bayesian analysis can be seen in Fig. 5b where, in this specific setting, the inset demonstrates that the Bayesian analysis allows for an order of magnitude reduction in the minimal detectable concentration.

### Micromolar range detection in the (sub)micron scale

Hyperdyne achieves excellent SNR with a relatively small number of measurements *N*_*m*_. The key question is whether such a diamond-based setup can combine high nuclear polarization with *M*_*z*_ Qdyne to achieve NMR spectrometry with chemical shift resolution on the nano-micro scale.

We propose the following setup for nano-micron scale NMR (Fig. 2(b)) - an analyte with *μ*M-mM concentration in a solution is placed on top of a nanostructured diamond for improved surface ratio, e.g. with nanoslits^47^. The walls of the ~0.3 *μm* wide nanoslits are assumed to be doped with NV centers to achieve a concentration of 10^17^ cm^−2^. We assume that the NV doping includes a micron-scale depth detection layer and a 5–20 nm near the surface layer. The Hyperdyne sequence is then composed of *N*_*m*_ alternations of hyperpolarization and *M*_*z*_ Qdyne sequence. The hyperpolarization, driven by the shallow NV centers, significantly enhances the measured signal.

Since NV centers can be optically polarized to over 90% polarization with microsecond-long laser pulses^48^, shallow NVs provide a unique resource for polarizing nuclear spins in nearby molecules. The polarization efficiency depends on ${g}_{tot}{\tau }_{c}$, where ${\tau }_{c}$ is the correlation time, and ${g}_{tot}=g\sqrt{{N}_{I}}$ is the total flip-flop coupling between the nuclear spins and the NV center, *N*_*I*_ the number of nuclear spins in the polarisation region, and *g* the average coupling to these nuclear spins. When ${g}_{tot}{\tau }_{c} < 1$, the typical scenario, the polarization efficiency does not depend on the analyte concentration^29^.

As the distance between slits is 300 nm, and with the chosen NV concentration the average distance between NV centers is 22 nm, to calculate the achieved polarization, on needs to consider the polarization buildup in a $22\,{\rm{nm}}\times 22\,{\rm{nm}}\times 150\,{\rm{nm}}$ region for each NV. Assuming ${g}_{tot}{\tau }_{c} < 1$, a nuclear ${T}_{1}\sim 2\,{\rm{s}}$, using robust polarization pulses^49^ and experimentally verified polarization transfer rates^29^, we obtain 0.5% polarization of the analytes in the nanoslit solution after 2 seconds of polarization, as shown in Fig. 5(a). the diffusion of the analyte is assumed to be around 10^−11^ m^2^/s, either due to large analytes (e.g. proteins) or a viscous solution. The polarization simulation takes into account the polarization rate, the nuclear relaxation process and the molecular diffusion.

For thermal polarization the duty cycle for the measurements is limited by the time required to build up the thermal polarization, on the order of several *T*_1_ times. Unsurprisingly, as the nuclear *T*_1_ is the limiting time also for the polarization buildup, replacing nuclear thermalization by NV-based DNP does not change the duty cycle of the NMR experiments.

The achieved NMR sensitivity now depends on the volume of material probed, as *N*_*NV*_ scales linearly with the volume (the solution is assumed to reside mainly in the nanoslits, but the diamond surface cross section can be enlarged). Figure 5(b) shows the detectable analyte concentration within 10 minutes of measurement time (taking into account the time for polarization and the experimental photon detection efficiency) when varying the probe size, for the achieved polarization of 0.5% in each hyperpolarization cycle, with spectral and Bayesian analysis. As a comparison, best achieved sensitivity for microcoil and cryogenic probe NMR are noted^47^. For NMR spectroscopy we see that for a volume of less than 1 femtoliter (1 nanoliter), detection with a few Hz resolution of mM (*μ*M) concentrations is feasible with Hyperdyne within 10 minutes, corresponding to $3\times {10}^{6}\,{\rm{spins}}\times \sqrt{{\rm{Hz}}}$ (${10}^{18}\,{\rm{spins}}\times \sqrt{{\rm{Hz}}}$), paving the way for applicable diamond-based NMR spectroscopy. Even with hyperpolarization, these regimes would not be possible with standard microcoils due to the *V*^1/4^ scaling. Note that at very low concentrations, additional noise will be produced by the spatial location of the individual number of nuclear spins at the NV detection region.

Regarding the minimal linewidth detectable by the setup we note that due to the small width of the slits (300 nm) the nuclear spins are in close proximity of the NV center sensors and may be subject to shifts of the nuclear Larmor frequency due to the Z-Z coupling with the NV centers. This leads to a broadening in high-resolution NMR which we calculate to be in the range of *γ*_*e*_Δ*B* ≈ 1 − 2 Hz. Here, the presence of NV centers on both sides of the slit improves the homogeneity of the signal, as the deviation of the Larmor becomes more homogeneous spatially, see Supplementary Information.

### Resolution of chemical shifts

**Figure 6:**
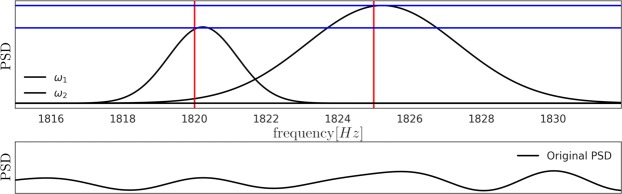
Comparison of detection results between Bayesian (upper part) and FFT (lower part) analysis of 160 *s* of simulated Toluene signal. The Bayesian analysis obtains the posterior densities of mean frequency positions, where the horizontal lines give the true value of the simulated signal. Further the heights of this peaks are scaled such that they correspond to the relative power contained in this resonance frequency.

To further underline the practical relevance of the Hypdyne scheme, we show that it is capable of resolving chemical shifts and therefore well suited for detection, spectroscopy and fingerprinting of molecules and compounds. To this end we generated atomistic scale simulations of the NMR signal of a toluene solution of 0.2 M concentration at 0.25 T field, whose detection was simulated for parameters typical of near surface NV-centers and realistic detection rates, see Supplementary Information. The upper part of Fig. 6 shows the result of a Bayesian data analysis of such a signal which is capable of inferring the central frequencies of the two NMR resonances of Toluene in the signal. The two NMR resonances, exhibiting a chemical shift of 5 *Hz* relative to each other, are found in our simulation at ${\omega }_{1}=1.820\,kHz$ and 1.825 *kHz*. Our Bayesian analysis of the photon cout record reveals several pieces of information, a probability density of the estimated frequency positions and from it the estimated central frequency as well as the relative power of the two NMR peaks. Both pieces of information, position and relative strength, provide the foundation for fingerprinting molecules. The lower part of Fig. 6 shows the result of a classical FFT analysis of the same photon count signal. This comparison clearly shows that application of Bayesian inference, which is making use of a priori knowledge about the underlying model leading to a measurement signal as well as sensible priors for system parameters outperforms uninformed FFT. It should be noted, that the 320 *s* of signal time used in the analysis could be equally acquired by a bulk of NVs concurrently. Similar to the case for a signal frequency for longer signal times, the relevant peaks will also become visible with FFT analysis.

## Discussion

The ability of NV centers to serve simultaneously as a hybrid quantum-classical detector and a source of hyperpolarization and to combine this with the analysis of the collected NMR signal with methods of signal processing based on Bayesian inference opens up new possibilities for (sub)micro scale NMR. It is interesting to note the uniqueness of the system - the NMR detection is based on individual electron spins, accumulating phase independently, very differently from other methods of high-field NMR detection (e.g. induction in tuned coils). This spin-based detection has similarities to the atomic-based magnetometry used in ZULF^10–13^, which has shown radically higher sensitivity in the zero-field regime compared to NMR coils. The use of NV centers as the electron detectors enable to expand this approach both to higher magnetic fields where atomic magnetometers are no longer usable, as well as the sub-micron scale (for both ZULF and high field). The fact that these same electron spins (at least the shallow ones) can also be optically polarized and serve as a source for suprathermal dynamic nuclear polarization for the investigated nuclear spins is a fortuitous coincidence for the hybrid polarizer/micro-NMR system, and enables achieving remarkable sensitivities. The achieved polarization of the molecules will depend on the molecular relaxation time and diffusion, and can be optimized for specific molecules.

Regarding the analysis of the *M*_*z*_ Qdyne signal, the introduced heirachical Bayesian analysis was shown to dramatically improve the detection sensitivity even in the regime where the central-limit theorem, typically use for NMR spectra analysis, does not apply. It is worth exploring how well this analysis could also improve non- hyperpolarized Qdyne, as it could push the limits (concentration, time, volume) of the regimes where it is applicable. Further it would be interesting, if inferred parameter combinations from signal with chemical shifts make it possible to do reliable fingerprinting of chemical compounds.

## Summary and Conclusions

In this work we have presented a blueprint for nanoscale NMR. Our approach builds on earlier work that demonstrates experimental feasibility of the required magnetic field detection scheme^25^, on theoretical and experimental work that developed and demonstrated polarization transfer from color centers to liquids^28–30^ and signal processing methods^39,45^.

## Methods

### Simulation parameters

All simulations were performed using a 6.1 nm or 12.2 nm deep NV center, with the diffusion coefficient of 10^−12^ *m*^2^/*s*. For the simulation of the toluene molecule, a 12.2 nm deep NV was used with 0.2 M concentration at 0.25 T magnetic field, using XY8-8 sensing sequences for the signal accumulations and a delay time of 64 ns between the pulses.

### Bayesian inference

In Bayesian statistics, a model of those aspects of the experiment that are relevant to the detected signal is built, and within this model parameters are considered as random variables. An estimation problem is then equivalent to the determination of the distribution of those parameters. For brevity of the manuscript, the model, parameters and algorithms are presented in detail in the Supplementary Information.

### Experimental parameters

The experiment was performed with the setup presented in^25^. The amplitude of the RF signal was calibrated by fitting the accumulated phase due to the statistical polarization to that measured by a 6.2 nm deep NV center^50^. For each run, the NV phase in each XY measurement was accumulated over 5.6 *μ*s, resulting in 1000 data points for the 5.6 ms long signal.

## Supplementary information

Supplementary Information
