# Supplementary material for: Blueprint for nanoscale NMR
Source: Sci Rep. 2019 May 6;9:6938. doi: 10.1038/s41598-019-43404-2 (PMC6502870; doi:10.1038/s41598-019-43404-2)
Supplement: Supplementary file 1 — Supplementary Information [file 41598_2019_43404_MOESM1_ESM.pdf]

Supplementary Material for Blueprint for nanoscale NMR

Ilai Schwartz[1,2], Joachim Rosskopf[1], Simon Schmitt[3], Benedikt Tratzmiller[1], Qiong Chen[1], Liam P. McGuinness[3], Fedor Jelezko[3], and Martin B. Plenio[1]

[1]Institute of Theoretical Physics and IQST, Albert-Einstein-Allee 11, Universität Ulm, 89081 Ulm, Germany
[2]NVision Imaging Technologies GmbH, Albert-Einstein-Allee 11, Universität Ulm, 89081 Ulm, Germany
[3]Institute of Quantum Optics and IQST, Albert-Einstein-Allee 11, Universität Ulm, 89081 Ulm, Germany

March 9, 2019

Classical NV Backaction Calculation

As our scheme makes use of many NV centers which impose a magnetic field onto the nuclei, it is important to check the induced broadening is smaller than the precision of few Hz we need to detect chemical shifts and J-couplings. As shown in [1], the problem can be reduced to simulating the dimensionless geometric factor

$$\int \mathrm{d}V \frac{1}{r^3} \left(3(\hat{r}\hat{u}_{NV})^2 - 1\right),$$

where r is the distance between a nuclear spin and the NV centers in the volume V over which is integrated, and u_{NV} is the NV orientation.

This integral was simulated for the geometry of nanoslits with alternately $300\mathrm{nm}$ of analyte and $200\mathrm{nm}$ of diamond in z-direction, and $6\mu\mathrm{m} \times 6\mu\mathrm{m}$ x-y area taking into account the two nearest layers. The geometric factor is quite homogeneous, with a standard deviation of < 0.6, and the main deviations origin from the spins on the edges of the considered $(3\mu\mathrm{m})^2 \times 300\mathrm{nm}$ volume, while in the center the NV field averages to almost 0.

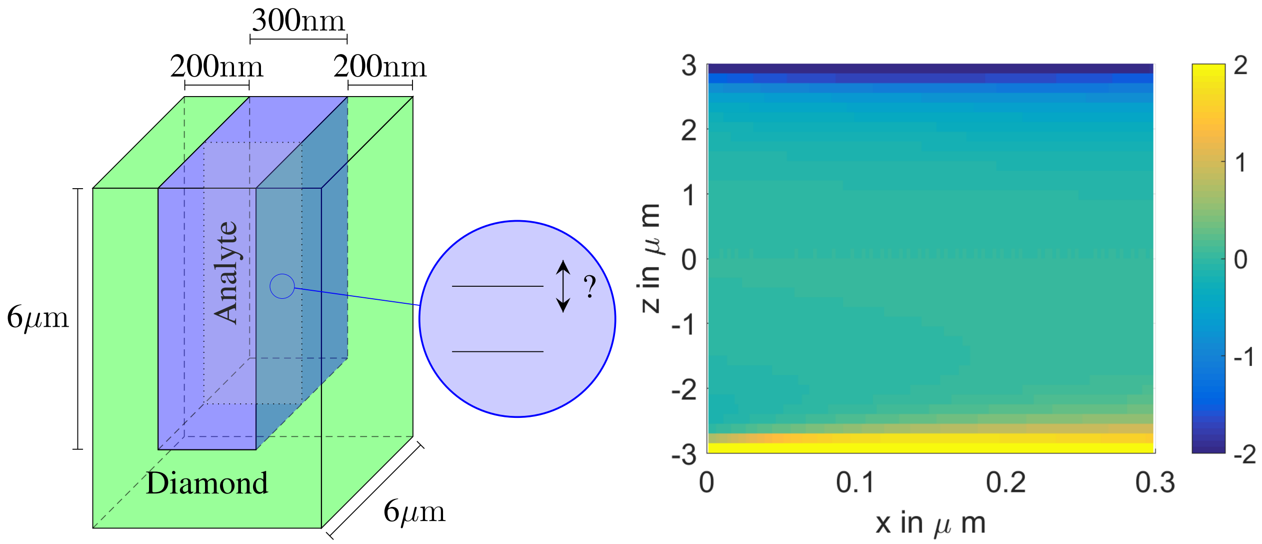

Figure 1: The setup of a nanoslit (left) can be used for our protocol, but we need to verify that the magnetic field from polarized NV centers left and right to the slit does not lead to significant broadening of the analyte two-level systems. The simulation of the geometric factor (right) shows that the broadening in the indicated plane (similar situation for the rest of the volume) is smaller than the shifts we want to resolve, in particular for the analyte with some distance to the NV surface.

This results in a broadening field of the order of $0.25\, nT$, leading to a broadening of the NMR line $< 1\, ppm$.

Backaction on nuclear spins due to NV measurements

The backaction from the measurements on the nuclear spins will decohere the nuclear spins for an infinite number of measurements, but it has a very limited effect in our case. The signal in equation (1) in the main text is a result of

measurements after interactions with the effective XY-8 Hamiltonian

$$H_{\text{eff}} = \sum_j \frac{2k_j}{\pi} \sigma_z \otimes \sigma_x^{(j)} \tag{1}$$

for a time τ_m, where j denotes the individual nuclear spins, σ_z ($\sigma_x^{(j)}$) denotes the NV (nuclear) pauli z (x) spin operator and k_j is the perpendicular coupling constant.

It can be shown analytically that the measurements decohere the nuclear spins as

$$\exp\left(-\frac{4k_j^2\tau_m^2}{\pi^2}N\right)$$

for N measurements.

As $1 \ll (k\tau_m/\pi)^2 = \sum_j k_j^2\tau_m^2/\pi^2$ is a condition for the measurement, this interaction is negligible when there are more nuclear spins involved than measurements done. In addition, spins diffusing in- and outside the detection volume also limit the effect of this backaction.

Description of the problem in terms of Bayesian statistics

In Bayesian statistics, a model of those aspects of the experiment that are relevant to the detected signal is built, and within this model parameters are considered as random variables. An estimation problem is then equivalent to the determination of the distribution of those parameters. For this purpose, the observed data is used to update the available prior information concerning the model parameters (also called prior or a priori knowledge and typically provided in the form of a probability distribution) to obtain the a-posteriori information (also known as posterior).

The examined distributions are described by densities p, which represent both the continuous and the discrete case, and are possibly multi-dimensional. Further let $p(\cdot,\cdot)$ be a joint and $p(\cdot|\cdot)$ a conditional distribution.

Given that $\boldsymbol{\theta} = (\theta_1, ..., \theta_l)^\top \in \mathbb{R}^l$ is a vector of unknown model parameters and $\boldsymbol{y} = (y_1, ..., y_n)^\top \in \mathbb{R}^{n\times m}$ are the data, $p(\boldsymbol{y}|\boldsymbol{\theta})$ is called likelihood of the data. The prior is given by $p(\boldsymbol{\theta})$ and we search for posterior density $p(\boldsymbol{\theta}|\boldsymbol{y})$. Using Bayes theorem, we can state that

$$p(\boldsymbol{\theta}|\boldsymbol{y}) \propto p(\boldsymbol{y}|\boldsymbol{\theta}) \cdot p(\boldsymbol{\theta}), \tag{2}$$

by neglecting a normalization constant. If not all parameters θ_i are of interest, we can split them into two groups

$$\boldsymbol{\theta} = (\boldsymbol{\theta}_{(1)}^\top, \boldsymbol{\theta}_{(2)}^\top)^\top, \boldsymbol{\theta}_{(1)} \in \mathbb{R}^{l_1}, \boldsymbol{\theta}_{(2)} \in \mathbb{R}^{l_2}, l = l_1 + l_2 \tag{3}$$

and one can state a posterior for the marginal density like

$$p(\boldsymbol{\theta}_{(1)}|\boldsymbol{y}) = \int_{\mathbb{R}^{l_2}} p(\boldsymbol{\theta}|y)d\boldsymbol{\theta}_{(2)} = \int_{\mathbb{R}^{l_2}} p(\boldsymbol{\theta}_{(1)}|\boldsymbol{\theta}_{(2)}, y)p(\boldsymbol{\theta}_{(2)}|y)d\boldsymbol{\theta}_{(2)}. \tag{4}$$

Often, a prior is not determined naturally and therefore has to be chosen suitably. This choice can have significant impact on the performance of the Bayesian inference, especially when the true values are in the margins of the chosen prior probability distributions.

Hierarchical Bayesian Models

Hierarchical Bayesian Models depend on special classification or arrangement of the parameters, e.g. $\boldsymbol{\theta} = (\boldsymbol{\phi}^\top, \boldsymbol{\psi}^\top)^\top, \boldsymbol{\phi} \in \mathbb{R}^k, \boldsymbol{\psi} \in \mathbb{R}^{l-k}$, such that the distribution of data $\boldsymbol{y}$ gets directly affected by $\boldsymbol{\phi}$, whereas $\boldsymbol{\phi}$ depends on $\boldsymbol{\psi}$:

$$p(\boldsymbol{y}, \boldsymbol{\phi}, \boldsymbol{\psi}) = p(\boldsymbol{y}|\boldsymbol{\phi}, \boldsymbol{\psi})p(\boldsymbol{\phi}|\boldsymbol{\psi})p(\boldsymbol{\psi}), \tag{5}$$

where one calls the parameters of the parameters $\boldsymbol{\psi}$ are called hyper parameters. This can of course be extended to deeper levels of parameter dependency hierarchies.

As the data is just affected indirectly by the hyper parameters, it follows that

$$p(\boldsymbol{\phi}, \boldsymbol{\psi}|\boldsymbol{y}) \propto p(\boldsymbol{y}|\boldsymbol{\phi}, \boldsymbol{\psi})p(\boldsymbol{\phi}, \boldsymbol{\psi}) = p(\boldsymbol{\psi})p(\boldsymbol{\phi}|\boldsymbol{\psi})p(\boldsymbol{y}|\boldsymbol{\phi}). \tag{6}$$

Therefore there has to be just priors chosen for the hyper parameters on the topmost hierarchy level. To describe the hierarchical models, which essentially form a graph of random variables, observed values and deterministic operations, we will further on rely on notation introduced in [8]. This notation gets especially interesting in the context of inference algorithms or probabilistic programs, which will be explained later and can be mapped directly onto implementation structure.

Markov Chain Monte Carlo (MCMC)

For a given Bayesian model consisting of likelihood and prior the actual calculation of integrals as in (4) is the essential problem. For a few limited models an analytical solution of the posterior is possible. Hence in general one has to rely on numerical methods to calculate the posterior $p(\boldsymbol{\theta}|\boldsymbol{y})$. In a Markov Chain Monte Carlo method, random variables are iteratively drawn

$$\boldsymbol{\theta}^{(t)} \in \mathbb{R}^l, t = 1, ..., T \tag{7}$$

from a density $\tilde{p}^{(t)}(\boldsymbol{\theta})$. Every sample just depends on the previous value, which is called the Markov property. The procedure is designed in such a way that the posterior is the stationary distribution of a Markov chain, i.e.,the distribution $\tilde{p}^{(t)}$ of the random numbers converges to the posterior. After a sufficiently long period, a sample approximates the remaining $\boldsymbol{\theta}^{(t)}$ well enough to derive higher statistical moments or do statistical tests on it.

Lets use a simple MCMC algorithm, the so called Metropolis-Hastings-Algorithm as an example. There one first draws a starting value $\boldsymbol{\theta}^{(0)}$ from a start distribution. Depending on that value the further $\boldsymbol{\theta}^{(t)}$ values are drawn, which are distributed due to a proposal distribution $\tilde{p}^{(t)}$. The proposal distribution has to be chosen that the support of the true posterior is completely covered and has roughly the same shape.

Given the values up to $\boldsymbol{\theta}^{(t)}$ one can draw a new candidate value $\boldsymbol{\theta}^*$ from $\tilde{p}^{(t+1)}$ and accept is as a new value from the chain with probability

$$\beta(\boldsymbol{\theta}^*|\boldsymbol{\theta}^{(t)}) = \min\left\{1, \frac{p(\boldsymbol{\theta}^*|\boldsymbol{y}) \cdot \tilde{p}^{(t+1)}(\boldsymbol{\theta}^{(t)}|\boldsymbol{\theta}^*)}{p(\boldsymbol{\theta}^{(t)}|\boldsymbol{y}) \cdot \tilde{p}^{(t+1)}(\boldsymbol{\theta}^*|\boldsymbol{\theta}^{(t)})}\right\}. \tag{8}$$

The posterior can be evaluated at the given points, since the normalization constants $p(\boldsymbol{y})$ in the quotient do cancel. With the acceptance probability defined one gets

$$\boldsymbol{\theta}^{(t+1)} = \begin{cases} \boldsymbol{\theta}^*, & \text{with probability}\beta \\ \boldsymbol{\theta}^{(t)}, & \text{with probability}(1-\beta) \end{cases}. \tag{9}$$

For details on convergence one can see Gelman et. al. 2014. Practically the performance of the sampling highly depends on it's initial starting value [7]. Therefore a maximum a posteriori (MAP) estimate is used as a starting point.

The step-size and direction are decided according to specific rules of the sampling method, including randomness (the Monte-Carlo aspect), gradient-seeking and momentum (Hamilton Monte Carlo [3]) for efficiency.

Recently tools for probabilistic programming (PP), automatic differentiation frameworks and advances in MCMC methods made automatic Bayesian inference on hierarchical models easy to formulate and perform. The tool used in this work is called PyMC3 [2]. The framework automatically derives a likelihood function for the model and repeats the sampling and evaluation for a defined upper bound. This reduces implementation effort and makes quick model changes possible.

Bayesian Parameter Estimation for Spectral Analysis

The above mentioned basic principles and terminology of hierarchical Bayes models and the parameter inference via MCMC is a starting point for doing spectral analysis. The measured Hypdyne photon count signal $\boldsymbol{y}$ is acquired by photo detectors. The detection scheme is similar to [4]. On the one hand the detection is extremely lossy, leading to a very sparse time series of photon counts. On the other hand each NV emits with a finite probability a photon in both the $|\uparrow\rangle$ and $|\downarrow\rangle$ state, leading to a just small net difference in detection probability r between ($r_{\uparrow} \approx 4.0\%$) and ($r_{\downarrow} \approx 2.5\%$). In the framework of Fourier NMR spectroscopy, this setting leads to quickly decaying SNR.

Under this challenging low SNR conditions, approximating the parameters of an underlying hidden model by Bayesian inference has shown great benefit in the analysis of various experimental data sources (e.g. in astro- or particle-physics [6, 5]). A Maximum likelihood method was applied successfully to a similar problem to recover central frequencies in [9]. Similar to FFT, the Bayesian method operates on the raw signal vector $\boldsymbol{y}$ without any preprocessing or reconstruction, but at the same time reducing the required signal to acquire by at least the order of magnitude. The method relies on a model which captures the hierarchical nature of the underlying oscillation, photon emission and detection process. This parametric model allows to incorporate prior knowledge of the problem into the analysis of the sparse signal. In the most simplest case, we analyze signals including a signal frequency.

Inference for a single frequency

By using Bayesian inference NMR, spectroscopy can be interpreted as fitting the distribution of parameters of an underlying harmonic model. The fit is guided by measured data $\mathbf{y}$ and an informed choice of priors of the parameters $\boldsymbol{\psi}$. At first we show how inference is done for a signal with spectrum exposing a single peak. In this case the parameter vector $\boldsymbol{\psi} = (g, \omega, \phi)$ is simpler than in the case of detecting a frequency shift, which will be shown in the next section. The hierarchical model used to infer the posteriors of the prior parameters is shown in figure 2. At it's core, there are two deterministic transformations. The first approximates the transition probability of an isolated NV-center

$$\boldsymbol{p} = \frac{1}{2} + \frac{1}{2}\sin(g\sin(2\pi\omega\boldsymbol{t} + \phi)), \tag{10}$$

while referencing the hyper parameters. The second relation

$$\boldsymbol{P} = N(r_{\downarrow} + (r_{\uparrow} - r_{\downarrow})\boldsymbol{p}), \tag{11}$$

represents the process of measuring the photon emission of N identical NV centers, with given detection probabilities $r_{\uparrow}$ and $r_{\downarrow}$.

With the actual measured values $\boldsymbol{y}$ clamped onto the model, we use MCMC inference to get the posterior distribution of the model's hyper parameters. If the MCMC algorithm has converged sufficiently well, the samples drawn approximate the respective posterior distributions of the random variables. The whole procedure can be seen as a

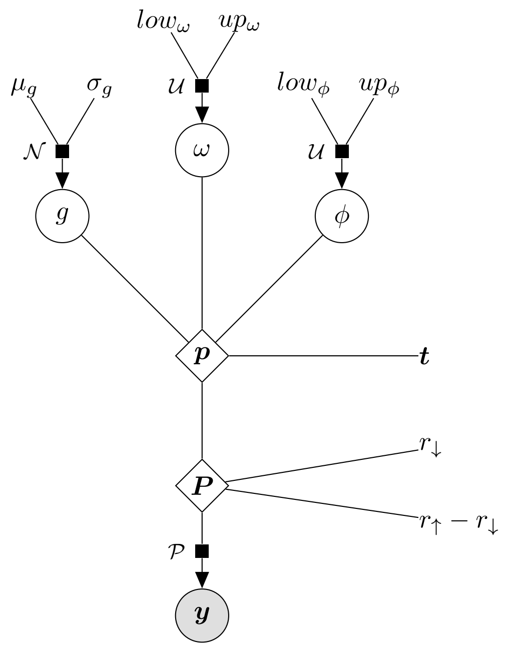

Figure 2: Hierarchical model for a single peak

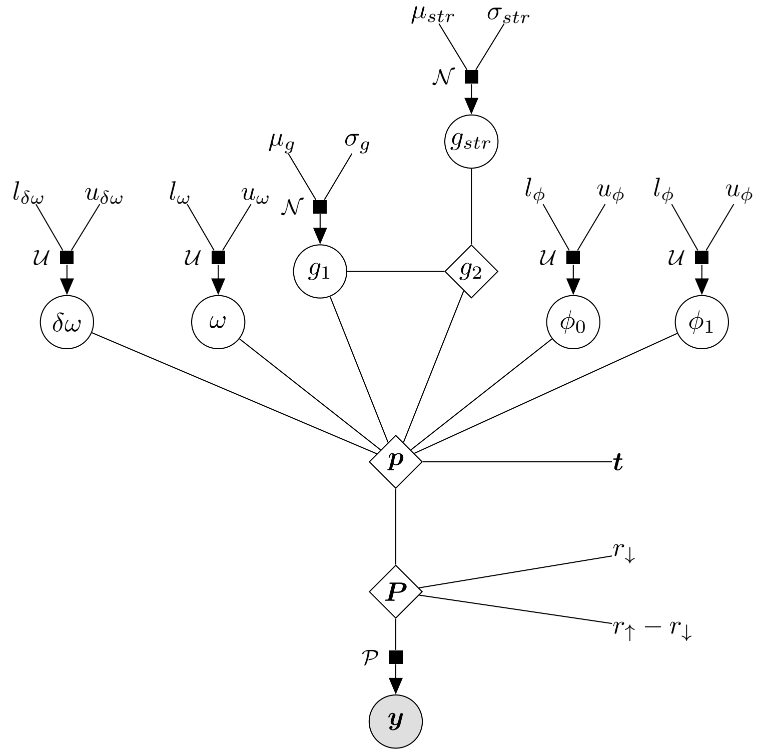

Figure 3: Hierarchical model for the shift between two peaks

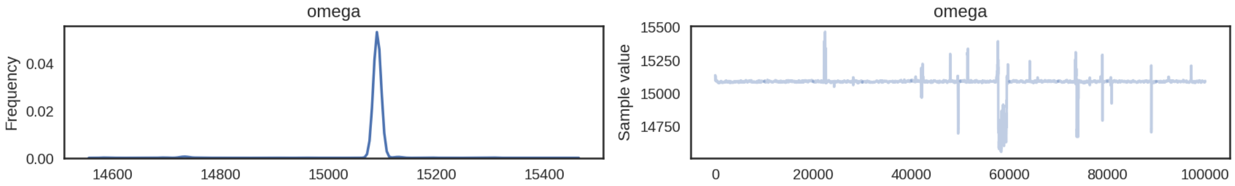

Figure 4: On the left, one can see a density estimate of the random variable ω sampled from the Markov Chain. On the right, the same data is shown as a so called ”trace”. There the frequency is drawn over the sequence of samples.

stochastic simulation of the experiment and adjustment of the parameters until measured and simulated data is statistically equivalent. Figure 4 depicts the result of this procedure. The more direct view into the method is shown on the right side, where the value of the random variable ω, which corresponds to the frequency. One can see, that the model pretty quickly converged to a single value, form which it deviates sometimes, but returns pretty quickly. In the same figure, on the left side, the same data is aggregated into a density, which shows the approximated posterior distribution of frequencies ω. Starting from a uniform distribution, this peaked posterior gives a good hint, that the model approximated pretty well, the underlying, latent frequency of the physical system. In our simulation the groundtruth frequency $\omega^* = 15.1\,kHz$.

Inference on frequency splitting

In a second step we extend the above model to describe a signal composed of two nearby frequency that exhibit a small splitting in frequency. The respective model diagram can be found in 3. The transition probability describes a beating between two (this can be extended to a higher order) frequencies. Therefore it takes the form:

$$\boldsymbol{p} = \frac{1}{2} + \frac{1}{2}\sin(\frac{1}{2}\cos(2\pi\omega_0\boldsymbol{t} + \phi_1)(g_1 + g_2\cos(2\pi\delta\omega\boldsymbol{t} + \phi_2 - \phi_1)) - \\ \frac{1}{2}g_2\sin(2\pi\omega_0\boldsymbol{t} + \phi_1)\sin(2\pi\delta\omega\boldsymbol{t} + \phi_2 - \phi_1)) \tag{12}$$

The inference is also done with an intelligently initialized MCMC algorithm. The posterior parameters are shown in 5. One can see, that there is a pretty good fit to the groundtruth hull frequency $\omega_0^* = 1.82\,kHz$ the shifted beating frequency of $\delta\omega^* = 7\,Hz$. The true relative weight of $(g_1/g_2)^* = 1.4$ is still well approximated.

Reconstruction of spectrum from frequency splitting

One has to note, that the inferred posterior parameters in the above described models, are not directly comparable to the spectra one gets from Fourier analysis on the raw photon count signal. Therefore we combined the information about ω_0, $\delta\omega$ and the relative weight (g_1/g_2) to reproduce a representation of the data comparable to a traditional power spectral density. The result can be seen in figure 6.

The figure shows the reconstruction result of the Bayesian method. In general the position of the central frequencies as well as their central frequencies are discovered reasonably well.

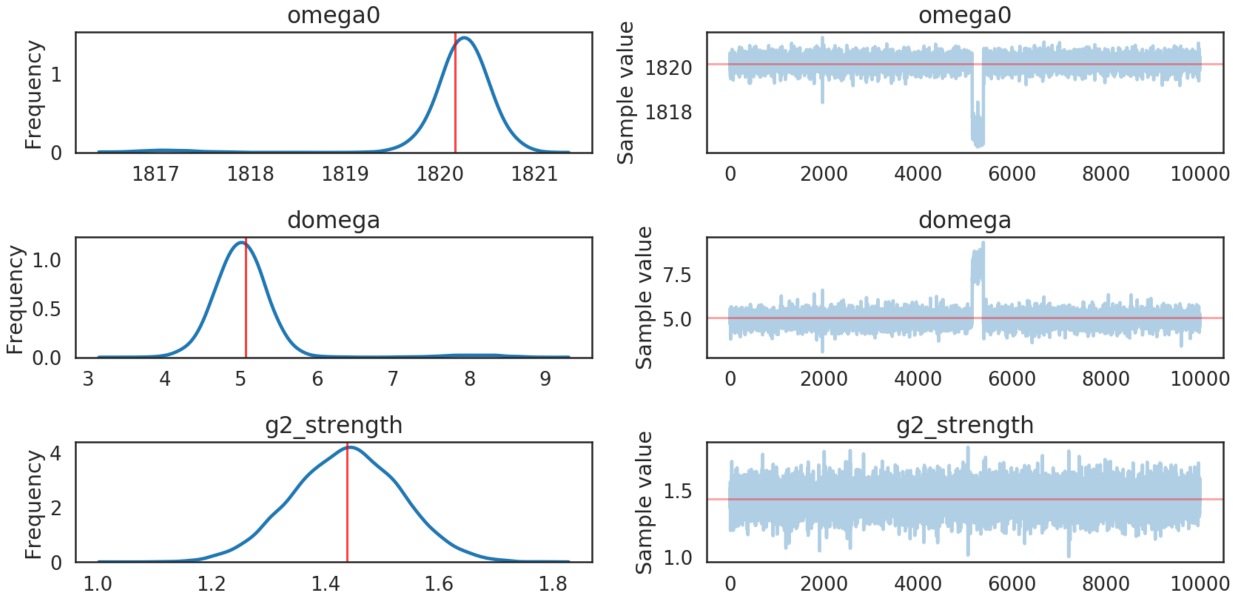

Figure 5: This figure is similar to 4. It shows the trace plots for the envelope frequency ω, the beating frequency $\delta\omega$ and the relative coupling strength $g_{2_{\text{strength}}}$.

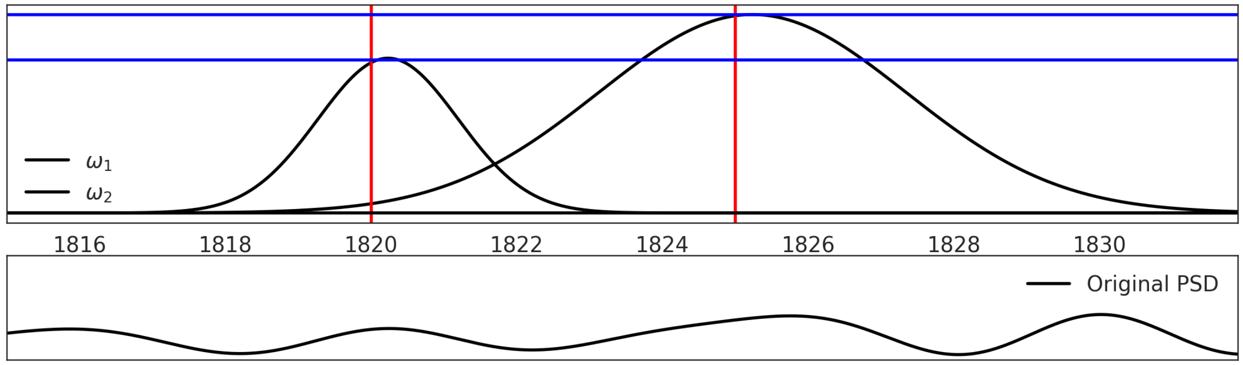

Figure 6: Combined information to make our result comparable to a traditional spectrum. The upper part of the figure shows the resulting two estimates of the central frequencies by the Bayesian inference process as black lines. The red vertical lines denote the groundtruth central frequencies. In the height of the estimates are scaled to be proportional to the inferred relative weight. The groundtruth for this quantity is depicted by the blue horizontal lines. The lower part of the figure shows, on the same frequency axis the PSD generated by FFT analysis directly on the photon count signal.

Simulation parameters

All simulations were performed using a 6.1 nm or 12.2nm deep NV center, with the diffusion coefficient of $10^{-12}\, m^2/s$. For the simulation of the toluene molecule, a 12.2 nm deep NV was used with 0.2M concentration at 0.25 T magnetic field, using XY8-8 sensing sequences for the signal accumulations and a delay time of 64 ns between the pulses.

At high NV and nitrogen concentrations electronic sources of noise become dominant, namely NV-P1 interactions and NV-NV interactions. We have verified in experiments that already at the relatively small magnetic fields concidered in this manuscript the XY8-N dynamical decoupling sequences decouple the NV centers from the P1 electron spins sufficiently, achieving over $30\, \mu s$ of coherence time. This is significantly longer than the XY8-8 sensing time used in the simulation ($\tau_m \sim 16\, \mu s$) . Though NV-NV interaction is not decoupled with these sequences, at the proposed concentrations the average distance between adjucent NV centers is around $20\, nm$, resulting in dipolar coupling $g_{NV-NV} < 10\, kHz$. As $\tau_m g_{NV-NV} < 1$ the NV-NV interaction will not cause significant decoherence in the measurement time-scale. If necessary, one could always decrease τ_m further to overcome other noise sources, resulting in a linear decrease in the SNR, according to Eq. 2 of the main text.

References

[1] D.R. Glenn, D.B. Bucher, J. Lee, M.D. Lukin, H. Park, and R.L. Walsworth, *High Resolution Magnetic Resonance Spectroscopy Using Solid-State Spins.* Nature **555**, 351 (2018).

[2] J. Salvatier, T. V. Wiecki, Ch. Fonnesbeck. *Probabilistic programming in Python using PyMC3.* PeerJ Computer Science 2 (2016): e55.

[3] M. Hoffman, A. Gelman. *The No-U-turn sampler: adaptively setting path lengths in Hamiltonian Monte Carlo.* Journal of Machine Learning Research 15.1 (2014): 1593-1623.

[4] T. Gaebel, M. Domhan, I. Popa, C. Wittmann, P. Neumann, F. Jelezko, ..., J. Meijer. *Room-temperature coherent coupling of single spins in diamond.* Nature Physics, 2(6), 408-413. (2006)

[5] F. Feroz, M. Hobson, M. Bridges. *MultiNest: an efficient and robust Bayesian inference tool for cosmology and particle physics.* Monthly Notices of the Royal Astronomical Society 398.4 (2009): 1601-1614.

[6] J. Hilbe, R. de Souza, E. Ishida. *Bayesian Models for Astrophysical Data: Using R, JAGS, Python, and Stan.* Cambridge University Press, 2017.

[7] J. Flegal, G. Jones *Implementing MCMC: estimating with confidence.* Handbook of Markov chain Monte Carlo, Boca Raton, Florida: Chapman & Hall/CRC, 175-197. (2011).

[8] Dietz, Laura. *Directed factor graph notation for generative models.* Max Planck Institute for Informatics, Tech. Rep (2010).

[9] Rotem, A., Gefen, T., Schmitt, S., McGuiness, L., Jelezko, F., Retzker, A. *Limits on spectral resolution measurements by quantum probes.* arXiv preprint arXiv:1707.01902. (2017).
